# Electroanalytical analysis of phenol oxidation using bacteria immobilized by a polycaprolactone coating on the copper electrode surface

**DOI:** 10.1038/s41598-024-58281-7

**Published:** 2024-06-07

**Authors:** Abdelaziz Moutcine, Charaf Laghlimi, Younes Ziat, Jalal Isaad, Soumia El Bahraoui, Abdelilah Chtaini

**Affiliations:** 1 Molecular Electrochemistry and Inorganic Materials Team, Faculty of Science and Technology, Sultan Moulay Slimane University, Beni Mellal, Morocco; 2 ERCI2A, FSTH, Abdelmalek Essaadi University, Tetouan, Morocco; 3 Engineering and Applied Physics Team (EAPT), Sultan Moulay Slimane University, Beni Mellal, Morocco; 4 Université du Québec à Chicoutimi, Chicoutimi, Canada

**Keywords:** Phenol oxidation, Cyclic voltammetry, Square wave voltammetry, Electrochemical impedance spectroscopy, Modified copper electrode, Analytical chemistry, Electrochemistry

## Abstract

The copper electrode modified by bacteria immobilised by a polycaprolactone film was successfully developed by electropolymerisation for the purpose of determining the presence of phenol. Electrochemical techniques such as square-wave voltammetry (SWV), cyclic voltammetry (CV) and electrochemical impedance spectroscopy (EIS) were used to characterize the electrochemical properties of the Cu-polymer/bacteria electrode. The results show that the intensity of the phenol oxidation peak increases with concentration, allowing us to obtain good analytical results with DL of 2.156 × 10^–7^ M and QL which is 7.2 × 10^–7^ M , confirming that the biosensor has excellent electroanalytical activity for phenol oxidation, with good stability and a wide linear range. Our electrode is based on a easily available and inexpensive material, as well as on its simple preparation, which has demonstrated high performance for phenol.

## Introduction

Phenol-based toxic organic compounds are commonly used in a wide variety of applications. Industrial activities such as the synthesis of macromolecules and resins, and the manufacture of pesticides, drugs and other phenol-based synthetic products, result in aqueous effluents polluted by toxic organic compounds^1–6^. On the other hand, phenols are poorly biodegradable, and their high toxicity leads to degradation of flora, fauna and aquatic life, as well as negative effects on human health^1,7–10^. Phenol can be life-threatening if ingested, if vapors are inhaled or if it penetrates the skin directly. It can cause severe burns and have adverse effects on organs such as the liver, lungs and nervous system^3,9–11^.

The methods of detection and decomposition of phenol are of great importance due to the danger it poses to the environment and to human health through diseases such as cancer.

A wide variety of techniques are currently used or have been developed for the remediation or degradation of phenols. These techniques include titrimetric^12,13^, spectrophotometric^14,15^, fluormetric^16^, chromatographic^17–20^.

Advances in nanotechnology have made it possible to study a wide range of advanced materials with special properties that have attracted considerable interest from researchers and led to applications in electrochemical sensors^21^. Recently, a variety of advanced materials of different sizes, with large active surfaces, good electrical properties and catalytic effects, have received particular attention and encouraged the development of sensing devices.

The electrochemical determination process involves converting the chemical quantity of the substance to be detected into an electrochemical reaction^22^. Commonly used working electrodes include the diamond electrode, the 3D-printed metal electrode, the graphene oxide electrode and glassy carbon electrodes modified with carbon nanotubes^23–26^.

The electrochemical sensor incorporating advanced materials is the most optimal sensing technology for rapid, sensitive and selective detection of phenolic contaminants. Phenolic contaminants are electrochemically active molecules that can be directly electrooxidised at appropriate potentials. Depending on the relationship between the response signal and the target concentration, rapid quantitative detection can be achieved. To date, several studies have been devoted to the detection of phenolic compounds. Ghalkhani et al. and Fernandes et al. have successfully investigated the detection of phenol and proposed oxidation mechanisms for phenolic compounds^27,28^, on graphene and conductive polymer materials, including phenol, 2-chlorophenol, 2,4-divhlorophenol, 2-nitrophenol, 2,4-dinitrophenol^24^.

The detection performance of the bare electrode is generally poor, so it is necessary to improve detection sensitivity by modifying the surface activity of the electrode. In this study, we prepared a biosensor by modifying the electrode surface using a simple electropolymerisation method to immobilise bacteria. The results obtained are encouraging for the electrode developed, which has significant electroanalytical activity for phenol oxidation and is easy to use.

As highlights of this study, the proposed sensor was easy to design, is simple and capable of detecting phenol directly in samples without simple special preparation, can be used on site and can be regenerated. Cyclic voltammetry and square-wave voltammetry were used as electrochemical detection techniques because of their sensitivity.

## Materials and methods

### Experiments

The electrochemical experiments were carried out in an electrochemical cell containing three electrodes connected directly to the potentiostat, Voltalab PGZ 100 (model PGZ 100, Eco Chemie B.V, Utrecht, The Netherlands), which was controlled by a computer allowing experimental data to be recorded and processed (Voltalab master 4 software). The working electrode was a 1 cm^2^ copper plate, supported by a copper rod to ensure current flow.

The working electrode surface was polished with p 4000 abrasive paper, cleaned with pure acetone and bi-distilled water, then immersed in a sulfuric acid solution for 5 min to remove any oxides formed on the surface.

The auxiliary electrode is a platinum plate with a surface area of 1 cm^2^ and the Ag/AgCl (3M KCl) electrode was used as a reference electrode. All three electrodes were immersed in a cell containing 1 M NaCl that used as the electrolyte solution and caprolactone monomer (5 ml)^29^, a polymer film developing on the surface of the copper plate during potential scanning.

The optimal pH for this study was chosen to be 5. The experiments were carried out in a cell with a volume of 100 ml of electrolyte solution containing 4 mmol of phenol. Cyclic voltammetry analysis was carried out between -3 V and 2 V at a sweep rate of 100 mV/s. Square wave voltammetry (SWV) was used under the following conditions: potential range −1.5 to 1.5 V, pulse amplitude 50 mHz and sweep rate 10 mV/s.

### Bacteria preparation

In this study, we have used the bacterial strain Staphylococcus aureus. It had been grown in Luria Burtani broth at 37 °C for 24 h after seeding. After being centrifuged for 15 min at 8400×*g*, subsequently washed twice with 0.1 M KNO_3_ solution, they were resuspended in the same solution. The physicochemical properties of the strain have been determined by contact angle measurements..

To ensure reproducible results, each experiment required the preparation of a new solution. The bacteria resuspended in the original solution obtained was diluted with water to obtain the required suspension at different concentrations before use.

### Reagents

All the reagents used in the electrochemical experiments were of analytical purity. They were used without any purification.

The chemicals we used were : NaOH and HCl were obtained from Sigma Aldrich. The standard solution was prepared from the phenol solution. This product was Fluka. The electrolytic medium was prepared using a sodium chloride (NaCl) solution from Sigma Aldrich. These solutions were prepared with bi-distilled water. The monomer used in this study is ε- caprolactone.

We controlled the acidity of the medium with a pH meter by adjusting with hydrochloric acid or sodium hydroxide.

### Analytical measurement

The proposed methodology was successfully applied to evaluate the potential of our electrode for practical analytical applications, the determination of phenol was carried out in tap water without any pre-treatment. 50 ml of a fresh sample was introduced into the cell and voltammetric measurements were carried out successfully.

### Ethics approval and consent to participate

All authors have read, understood, and have complied as applicable with the statement on “Ethical responsibilities of authors” as found in the Instructions for Authors.

## Results and discussion

### Phenol oxidation ability on a bare copper electrode

The working electrode was immersed in a 1 M NaCl electrolyte solution. The range of solvent and electrolyte stability was established by performing 10 cycles of cyclic voltammetry on the plate, stabilizing the plate surface (working electrode).

**Figure 1:**
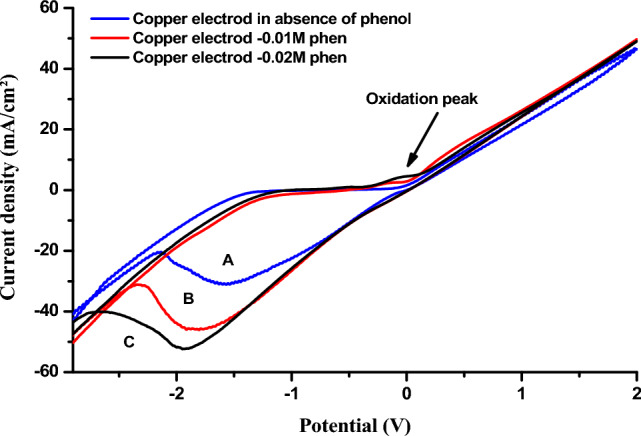
Cyclic voltamograms obtained by copper electrode (**A**) in the absence (**B**) in the presence of 0.01 M phenol, (**C**) 0.02 M phenol in 1 M NaCl (pH 5) at a scan rate of 100 mV s^−1^.

**Figure 2:**
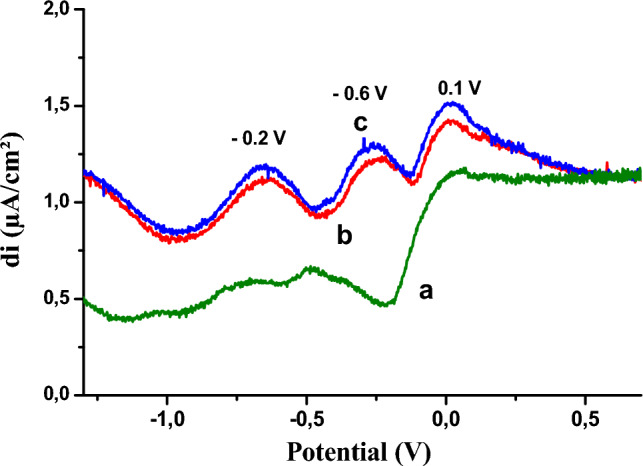
Square-wave voltamograms obtained by copper electrode (**a**) in the absence of phenol and (**b**) in the presence of 0.01 M phenol, (**c**) 0.02 M.

The oxidation capacity of phenol on the copper electrode surface was studied by cyclic voltammetry (VC) (Fig. 1) and square wave voltammetry (SQW) (Fig. 2).

As shown in Fig. 1, we present the voltammograms recorded at the surface of the Cu electrode in the absence and presence of different phenol concentrations.

It can be seen that the presence of phenol in the electrolyte solution leads to the appearance of a low-intensity oxidation peak around −0.1 V, which increases with phenol concentration and can be attributed to phenol oxidation. In the cathodic sweep direction, the copper reduction peak is due to the presence of phenol.

The behavior of the copper electrode towards phenol oxidation has been studied by SWV (Fig. 2). This method presents the advantage of neglecting the capacitive current and measuring only the faradic current resulting from electrochemical reactions, thus increasing the electrode's sensitivity.

We have observed two reduction peaks at −0.2 V and −0.6 V, which increase with phenol concentration, while an oxidation peak appears at around 0.1 V, which also increases with phenol concentration.

**Figure 3:**
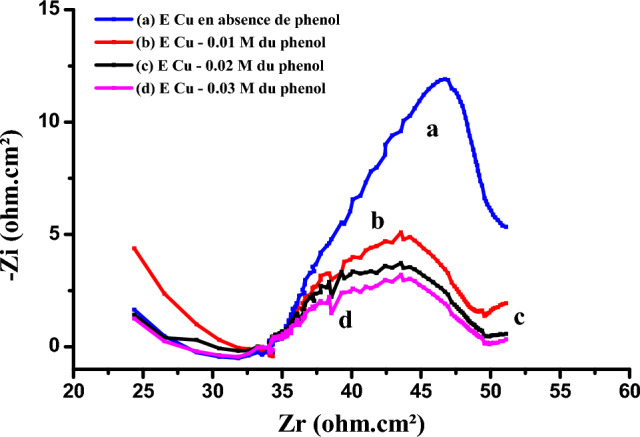
Cu- electrode impedance diagrams: (**a**) in the absence of phenol (**b–d**) in the presence of phenol 0.01 M; 0.02 M; 0.03 M.

We have used the electrochemical impedance on the Nyquist diagram (Fig. 3) as confirmation of these results. The diagrams are in the form of half-loops, the diameter of which corresponds to the electrode's resistance to electron exchange with the electroactive species; this resistance to charge transfer decreases in the presence of phenol.

Based on electrochemical results, our copper electrode can be used as an electrochemical biosensor.

### Effects of immobilizing bacteria on the copper electrode surface

**Figure 4:**
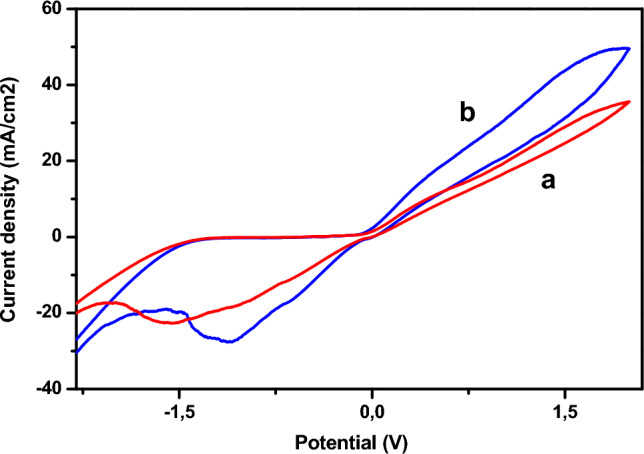
Cyclic voltamograms obtained by electrode of (**a**) copper Cu, (**b**) Cu-bacteria, in 1 M NaCl (pH 5), 100 mV s^−1^.

Figure 4 illustrates the cyclic voltammograms recorded respectively for the Cu and Cu-Bacteria electrodes after 20 min of contact between the electrode and suspended bacteria, in 1 M NaCl electrolyte medium and at a sweep rate of 100 mV s^−1^. It can be seen that the current density increases after immobilization of the bacteria on the electrode surface, as a result of the fact that the presence of bacteria on the electrode surface increases its activity. On the other hand, the presence of bacteria does not alter the cyclic voltammogram, showing that bacteria do not change the electrochemical characteristics of the electrode.

#### The effect of bacteria on phenol oxidation on the Cu electrod surface

We have suggested using bacteria as biocatalysts for the activation of phenol oxidation.

**Figure 5:**
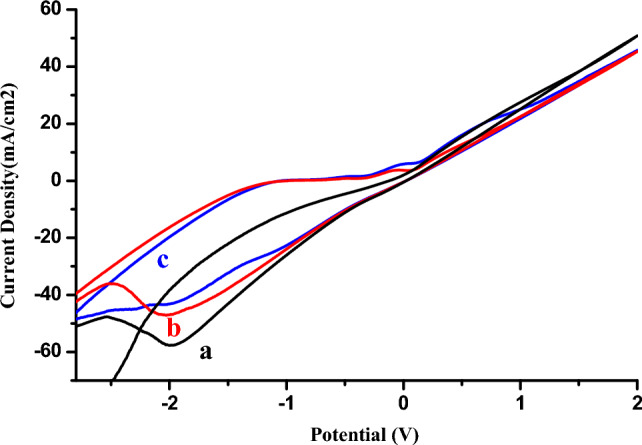
Cyclic voltamograms obtained by copper electrode (**a**) in absence of phenol, copper electrode (**b**) and Cu-bacteria electrode (**c**) in the presence of phenol in 1 M NaCl (pH 5), 100 mV s^−1^.

**Figure 6:**
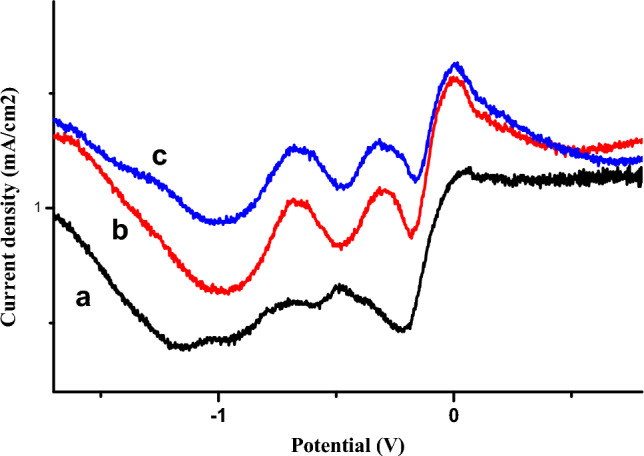
Square-wave voltamograms obtained by the copper electrode (**a**) in absence of phenol, copper electrode (**b**) and the Cu-bacteria electrode (**c**), in the presence of phenol in 1 M NaCl (pH 5), 50 mV s^−1^.

Figures 5 and 6 present, respectively, the cyclic and square voltamograms recorded for the copper electrode (a) in absence of phenol, the copper electrode (b) and the Cu-bacteria electrode (c) in the presence of phenol, in NaCl electrolyte medium, at a sweep rate of 100 mV s^−1^.

Indeed, Figs. 5 and 6 illustrate a comparative analysis of the influence of bacteria on the electrode surface in an electrolytic medium containing 4 mmol l^−1^ phenol. The bare copper electrode shows a modest change in current density in the presence of phenol compared with the same electrode in the absence of the analyte, but following immobilisation of the bacteria, the current density again increases in the presence of phenol without destroying the shape, so these results demonstrate that the presence of bacteria has a positive effect on the response of the electrode.

This would suggest that the bacteria have a very significant activity in catalyzing phenol oxidation, and for this reason we have proposed a relationship by which we can calculate the bacterial activity immobilized on the surface of the elaborated electrode (Eq. 1).

1 $$\alpha =\left(|1-\frac{Ibac}{I}|\right)*100$$

Based on the square-wave voltammograms, we have used the oxidation current peak to calculate bacterial activity at the electrode surface as follows:

$$\alpha =\left(|1-\frac{1.45}{0.95}|\right)*100 = 52.63\mathrm{\%}$$

**Figure 7:**
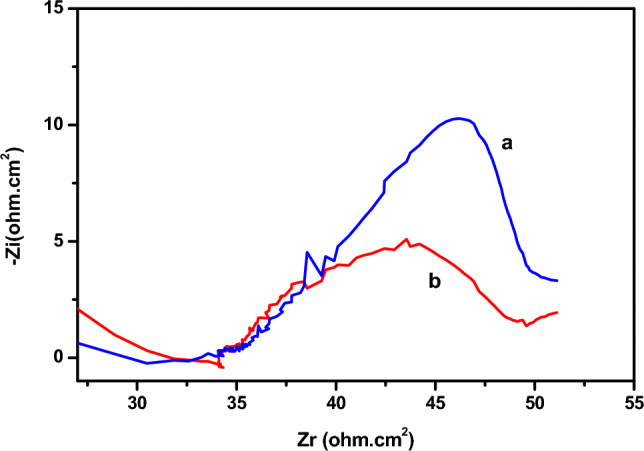
EIS diagrams of the Cu-electrode (**a**) and the Cu-bacteria electrode (**b**), in the presence of phenol.

Figure 5 shows the cyclic voltamograms recorded respectively for the Cu (a) and Cu-bacteria (b) electrodes in the presence of phenol, at a sweep rate of 100 mV.s-1. It can be seen that the presence of bacteria on the surface of the copper electrode leads to an increase in current densities, which are particularly visible in the SWV (Fig. 6). The above results are confirmed by impedance measurements (Fig. 7), in which a decrease in half-loop diameter can be seen at high frequencies, indicating a decrease in charge transfer resistance. These results suggest that the bacteria catalyzed the electrochemical oxidation of phenol.

#### Proposed reaction mechanism for phenol electro-oxidation

**Figure 8:**
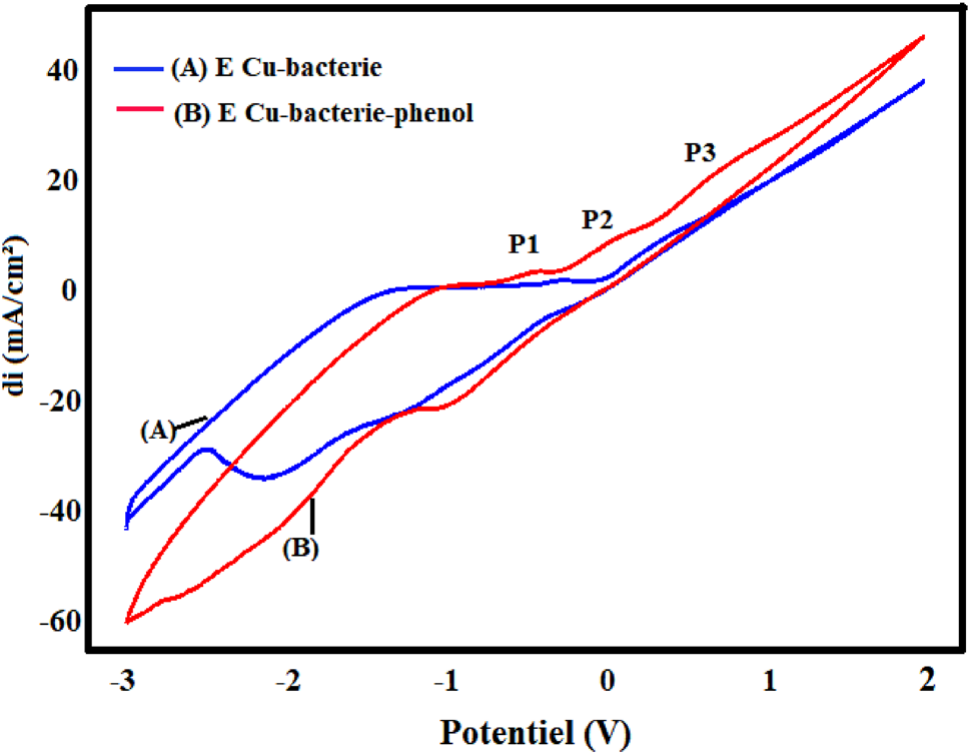
Cyclic voltamograms obtained by Cu-bacteria electrode (**A**) absence of phenol (**B**) presence of phenol in 1 M NaCl (pH 5), 100 mV s^−1^.

Phenol electro-oxidation by the Cu-Bacteria biosensor was carried out by cyclic voltammetry, at a scan rate of 100 mV.s^-1^ in a 1M NaCl electrolyte solution. Figure 8 illustrates the cyclic voltammograms recorded in the presence and absence of phenol for the bacteria-modified copper electrode (Cu-Bacteria).

**Scheme 1:**
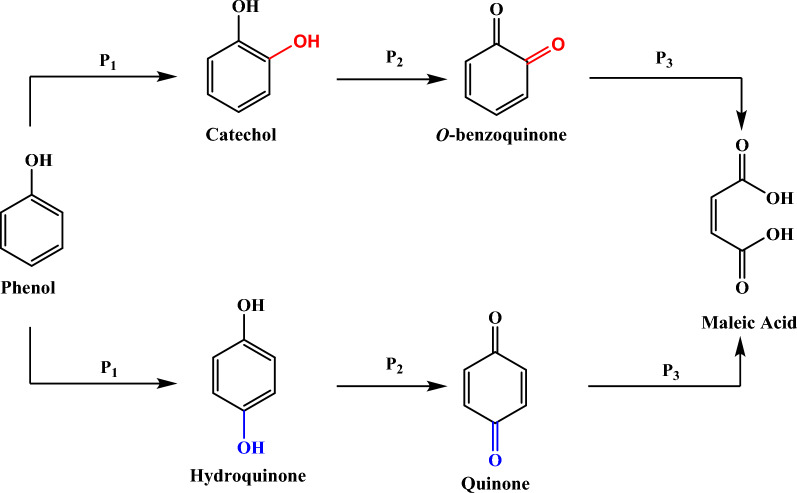
Proposed reaction mechanism for oxidation of phenol.

We have observed the appearance of 3 anodic peaks, at -0.43V, 0.07V and 0.71V respectively, due to the oxidation of phenol and its derivatives, for which we have proposed the following mechanism in Scheme 1:

We have considered these successive reactions:

The first electrochemical oxidation reaction of phenol (P1) takes place to hydroquinone or catechol. Electron transfer occurs at the working electrode when a positive potential is applied, and phenol is the electron source in this process. This reaction involves the loss of electrons from phenol to form hydroquinone or catechol. In the second reaction, one of the two elements, hydroquinone or catechol, is oxidised to quinone and a-benzoquinone respectively. Electron transfer occurs again at the working electrode, but this time it is the hydroquinone or catechol that loses electrons, resulting in the formation of quinone and a-benzoquinone. The quinone or a-benzoquinone can then undergo a third oxidation reaction to produce maleic acid (C_4_H_4_O_4_). The reaction to convert the quinone or a-benzoquinone into maleic acid again involves the loss of electrons, which transforms the quinone/a-benzoquinone into a more complex species, namely maleic acid.

### Modification of the copper electrode surface by a polymer

The poly-ε-caprolactone film has been electrochemically developed, in situ, on the surface of the copper electrode.

For the determination of the solvent and electrolyte stability range, 10 cycles of cyclic voltammetry have been performed on the copper electrode in order To form the film on the electrode surface. This resulted in an increase in the intensity of the polymerization peak.

We have used electrochemical polymerization for its ease of use and its ability to immobilize enzymes and bacteria on the electrode surface. Electro-polymerization was carried out using a solution containing caprolactone monomer and bacteria, resulting in the deposition of a thin film on the electrode surface.

**Figure 9:**
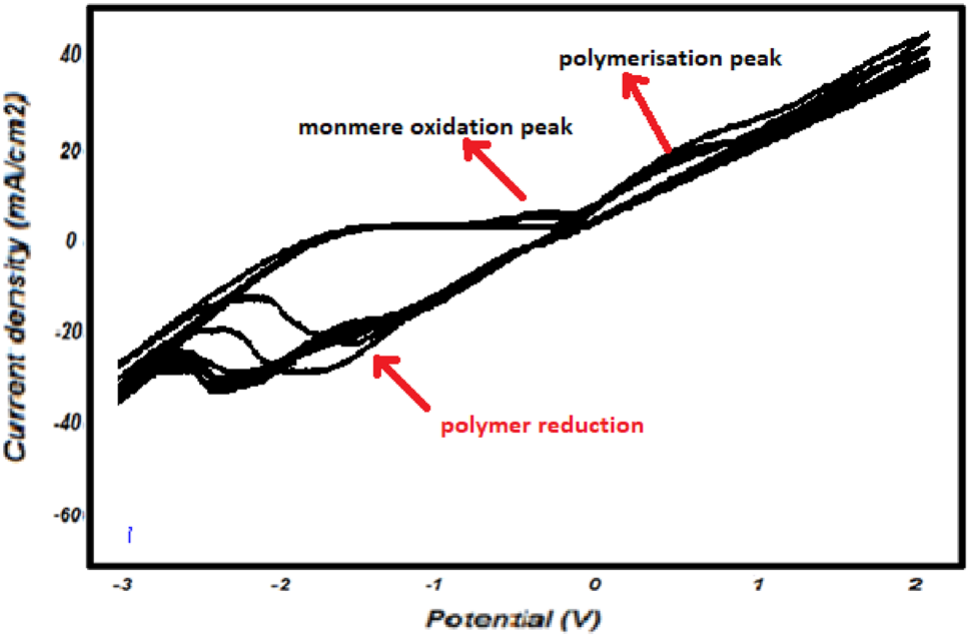
Cyclic voltamograms of monomer electro-polymerization and poly-ε-caprolactone film formation on the copper surface in 1 M NaCl (pH 5), 100 mV s^−1^.

As shown in Fig. 9, the appearance of a monomer oxidation peak and a polymerization peak, which increase with the number of cycles, indicates that the electrode surface is being modified.

**Figure 10:**
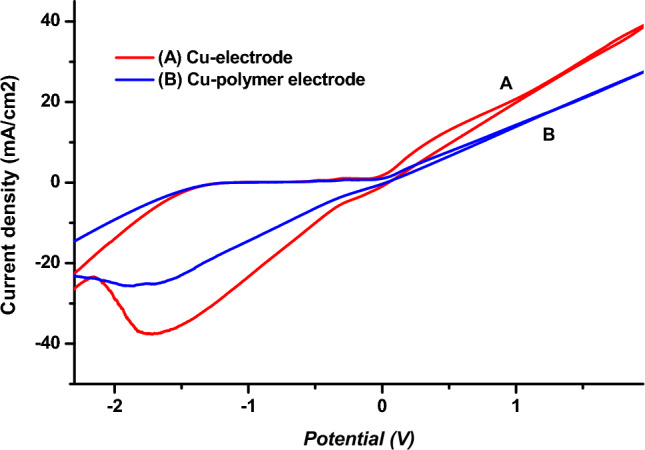
Cyclic voltamograms obtained by the Cu electrode before (**A**) and after (**B**) polymerization of ε-caprolactone monomer in 1 M NaCl, at a scan rate of 100 mV s^−1^.

In Fig. 10, cyclic voltammograms have been shown for copper (A) and copper- poly-ε-caprolactone (B) electrodes, respectively, in a 1 M NaCl electrolyte at 100 mV s^−1^. As can be seen, the voltammograms show different patterns accompanied by a decrease in current density after polymerization, suggesting that the surface of the Cu-polymer electrode is indeed modified by the poly-ε-caprolactone film.

#### Oxidation of phenol on the surface of polymer-modified copper

**Figure 11:**
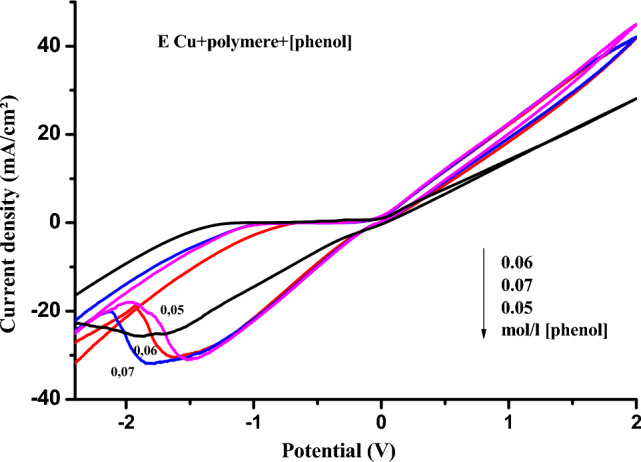
Cyclic voltamograms of the effect of phenol concentrations (from 0.05 to 0.07 M) in 1 M NaCl (pH 5) on the Cu electrode modified with poly-ε-caprolactone, V = 100 mV s^−1^.

It can be seen that the phenol oxidation current density peak on the polymer film-modified copper electrode increases with increasing phenol concentration, while the phenol reduction peak changes in the same way with increasing phenol concentration (Fig. 11).

#### Bioelectrode design and characterization

As described above, an electrode of copper is polymerized in a solution containing bacteria in suspension, during a contact time of 20 min.

**Figure 12:**
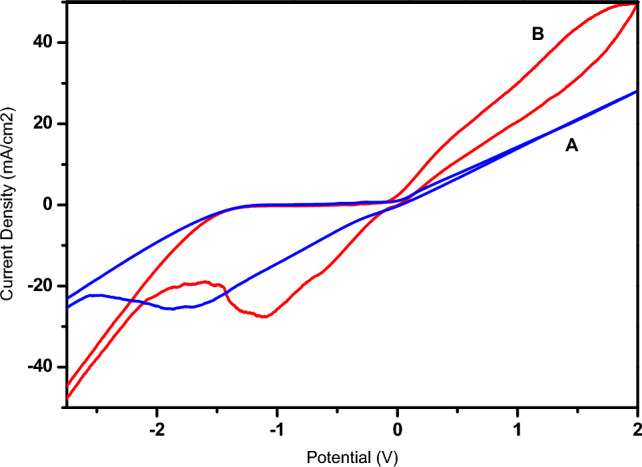
Cyclic voltamograms obtained by copper-poly-ε-caprolactone electrode (**a**) and Cu-poly-ε-caprolactone-bacteria electrode (**b**), in 1 M of NaCl (pH 5), 100 mV s^−1^.

Figure 12 illustrate, respectively, the cyclic and square voltammograms recorded on Cu-polymer (A) and Cu-polymer-bacteria (B) electrodes, in NaCl (1M) electrolyte medium at a scan rate of 100 mV.s^-1^. As can be seen, the presence of bacteria on the surface of the Cu-polymer electrode increases current densities.

#### Electrooxidation of phenol on the surface of the Cu-poly-ε-caprolactone /bacteria electrode

**Figure 13:**
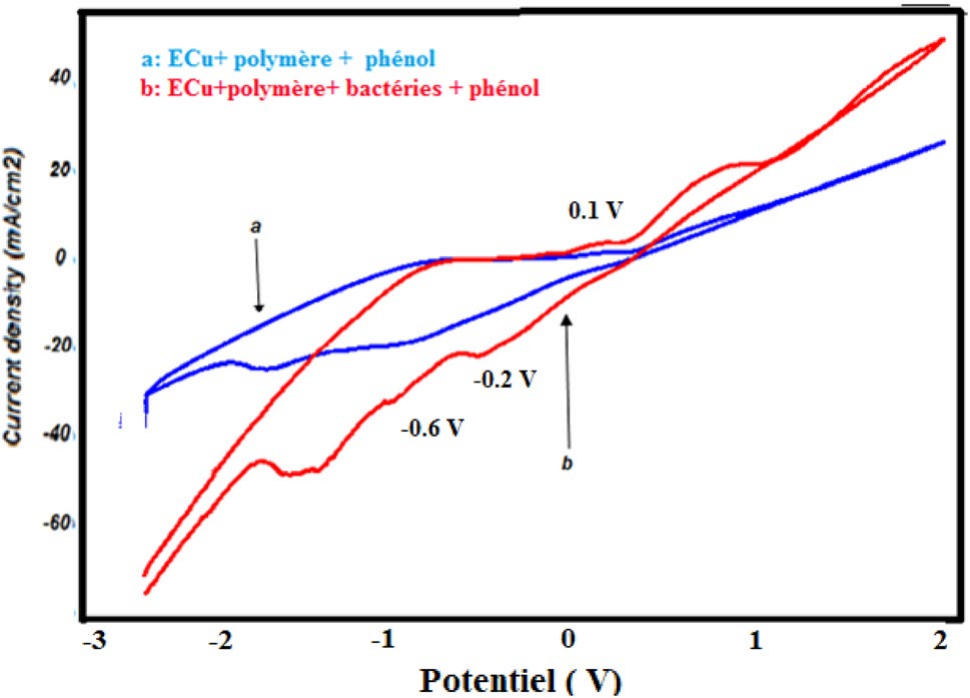
Cyclic voltamograms obtained by copper electrode modified by poly-ε-caprolactone (**a**) and Cu-poly-ε-caprolactone-bacteria (**b**) in the presence of phenol in 1 M NaCl (pH 5), 100 mV s^−1^.

**Figure 14:**
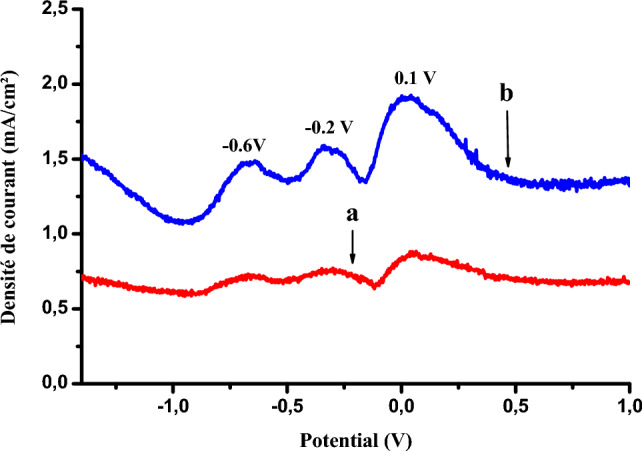
Square-wave voltamograms obtained by Cu-poly-ε-caprolactone (**a**) and Cu-poly-ε-caprolactone-bacteria (**b**) electrodes in the presence of phenol in 1 M NaCl (pH 5), 50 mV s^−1^.

The electro-oxidation of phenol on the Cu-poly-ε-caprolactone -bacteria biosensor has been studied using cyclic voltammetry (Fig. 13) and square-wave voltammetry (Fig. 14).

Figure 13 shows the cyclic voltammograms recorded by the Cu- poly-ε-caprolactone electrode (curve a) and the Cu- poly-ε-caprolactone-bacteria electrode (curve b), respectively, in an electrolytic medium containing 0.01 M phenol.

It can be seen that the presence of bacteria leads to the appearance of three peaks: an oxidation peak at around 0.1 V and two reduction peaks at −0.2 V and −0.6 V, meaning that the current density of the peaks are remarkably consistently high.

It can be said that the immobilisation of bacteria on the surface of the Cu-poly-ε-caprolactone electrode has a positive effect on the activity of the electrode with regard to the oxidation of phenol, allowing an acceleration of the transformation of phenol to a more advanced stage (peak oxidation). These results were confirmed by square-wave voltammetry, as shown in Fig. 14.

### Calibration curve

**Figure 15:**
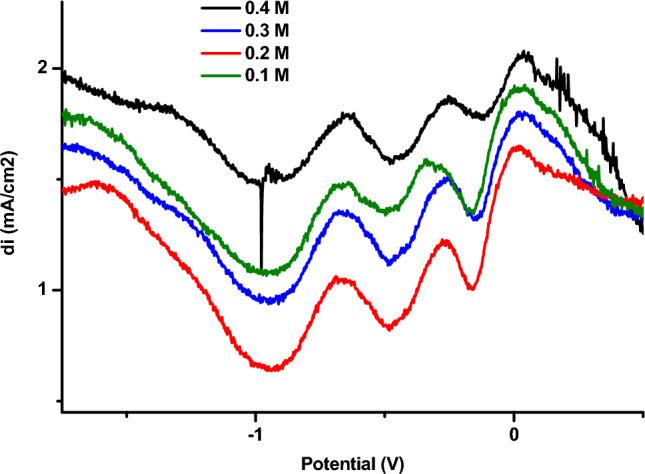
Square-wave voltamograms recorded for the copper electrode modified by poly-ε-caprolactone/bacteria. Effect of phenol concentration, 0.01–0.04 M—50 mV s^−1^.

**Figure 16:**
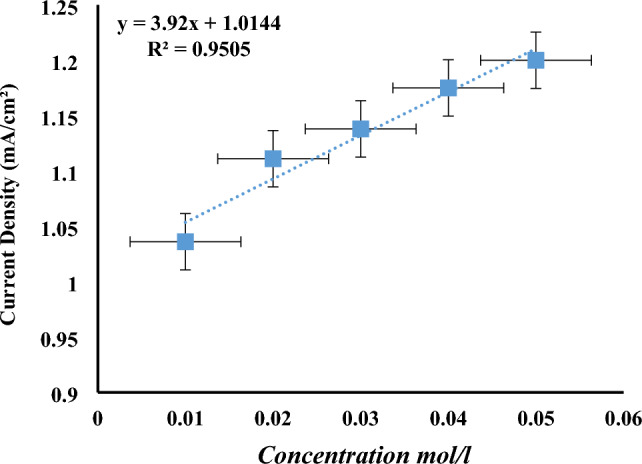
Influence of phenol concentration on the intensity of oxidation peaks obtained by SWV at the surface of the copper-poly-ε-caprolactone/bacteria electrode.

The variation in cathodic peak intensity as a function of phenol concentration has been followed using square-wave voltammetry (Fig. 15). The calibration curve has been established by gradually adding phenol to the electrolyte solution (for concentrations between 0.01 and 0.05 M (Fig. 16). As can be seen, the oxidation current peaks increase linearly with phenol concentration. This linearity can be expressed by the following relationship.

According to the square voltammograms, increasing phenol concentration leads to an increase in oxidation peaks. The intensity of these peaks have been used to plot the calibration line as shown in Fig. 16.

Analytical parameters, in particular the limit of detection, standard deviation and limit of quantification, were determined from this curve. These values were determined using the Miller-Miller method.

The limits of detection and quantification are 2.156.10^–7^ M and 7.2.10^–7^ M respectively.

The electroanalytical method was used to detect phenol in real samples such as water. The proposed electrode proved highly sensitive for phenol detection (2.156.10^–7^ M).

**Figure 17:**
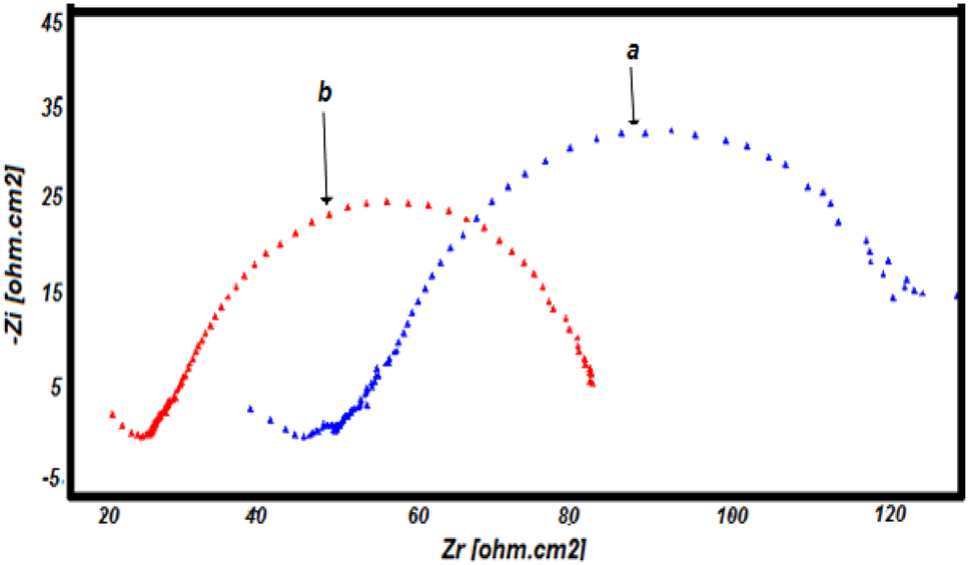
Diagrams of impedance of polymer-modified copper (**a**) in the presence of phenol (**b**) bacteria in the presence of phenol.

The electrochemical parameters obtained from these diagrams (Fig. 17) are presented in Table 1.

**Table 1:** Electrochemical impedance parameters (R1 being the electrolyte resistance and R2 the transfer resistance, and C the double layer capacitance).

	R_1_ (Ω cm^2^)	R_2_ (Ω cm^2^)	C (mF/cm^2^)	Diameter (Ω cm^2^)
Cu-poly-phen	55.14	110.3	1.026	109.6
Cu-poly-bact-phen	35.85	72.26	2.2	71.87

The diameter of the capacitive circuit decreases in the presence of phenol and bacteria, which is explained by the decrease in charge transfer resistance. The capacitance of the double layer, which models the effect of the drop in potential near the electrode, increases in the presence of bacteria and phenol, these is due to the significant oxidation of phenol on the surface of the copper electrode modified by poly-ε-caprolactone.

## Conclusion

In this study, we have demonstrated the preparation of a copper-based electrode. The bare copper surface is not actively involved in phenol oxidation, but can be modified by immobilizing bacteria. The aim of this work was to bring together two methods of phenol destruction: the electrochemical method, often blocked by poisoning of the electrode surface through the formation of reaction intermediates, and the biological method based on phenol biodegradation by bacteria. The new method of immobilizing Staphylococcus bacteria on a copper electrode modified with a poly-caprolactone film has been introduced.

The proposed electrode was shown to perform satisfactorily for the detection and oxidation of phenol in the proposed concentration range, with an LOD of 2.156.10^–7^ M and a QL of 7.2.10^–7^ M.

Based on these results, we have investigated new polymer-modified electrodes for water quality monitoring. The simplicity and reproducibility of the polymer-modified electrodes represent a promising new technique for the detection of trace levels of organic pollutants.

## Data Availability

The datasets used and/or analysed during the current study available from the corresponding author on reasonable request.
